# Supplementary figures and images for: Mechanisms of multi-species mealybug invasions in Hainan Island of China: Integrating niche, distribution, and habitat drivers
Source: PLoS One. 2025 Oct 24;20(10):e0333679. doi: 10.1371/journal.pone.0333679 (PMC12551864; doi:10.1371/journal.pone.0333679)

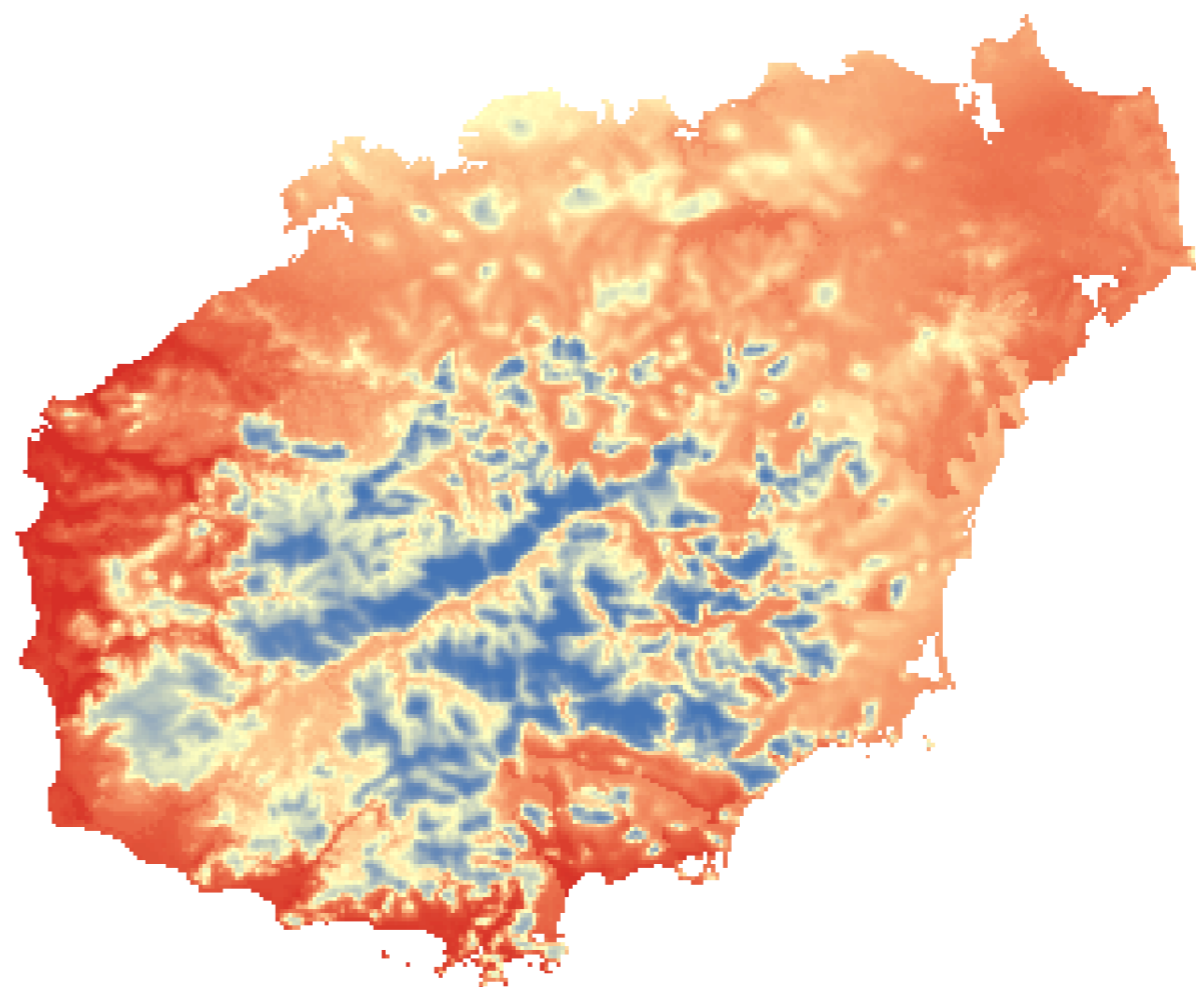

Supplement: S2 Fig — (PDF) [file pone.0333679.s002.pdf]
